# microRNA-670 modulates *Igf2bp1* expression to regulate RNA methylation in parthenogenetic mouse embryonic development

**DOI:** 10.1038/s41598-020-61816-3

**Published:** 2020-03-16

**Authors:** Jindong Hao, Haobo Hu, Ziping Jiang, Xianfeng Yu, Chengshun Li, Lin Chen, Yidan Xia, Da Liu, Dongxu Wang

**Affiliations:** 1 Laboratory Animal Center, College of Animal Science, Jilin University, Changchun, China; 2 Department of hand surgery, The First Hospital of Jilin University, Changchun, China; 3 Department of Pharmacy, Changchun University of Chinese Medicine, Changchun, China

**Keywords:** Developmental biology, Embryology

## Abstract

Aberrant epigenetic modification, including *N*^6^-methylation of adenosine (m6A), has been frequently reported in embryos derived from parthenogenetic activation (PA). However, the role of *Igf2bp1* expression pattern in m6A modification and the mechanism through which *Igf2bp1* function is regulated in PA embryos remains elusive. Therefore, in this study, using si-Igf2bp1 and betaine (*N,N,N*-trimethylglycine, a major methyl donor), we investigated the effect of *Igf2bp1* expression in m6A modification on the development of PA embryos. The results indicated that the down-regulation of *Igf2bp1* reduced the cleavage and blastula rates of PA embryos. Moreover, m6A expression level was markedly down-regulated following microinjection with si-Igf2bp1. However, the treatment with betaine could significantly restore the m6A level. Further bioinformatics analysis revealed *Igf2bp1* as the putative target of microRNA 670 (miR-670). Thus, to confirm this finding, mimics and inhibitor of miR-670 were microinjected into PA embryos. The results demonstrated that miR-670 inhibitor augmented the expression of *Igf2bp1* and rescued cleavage and blastula rates. In addition, the miR-670 inhibitor promoted the m6A expression level. TUNEL assay revealed a loss of expression of *Igf2bp1* induced cell apoptosis in PA embryos. Taken together, these results demonstrated that miR-670-3p functions as the regulator of *Igf2bp1* expression and plays a crucial role in PA development through m6A modification.

## Introduction

Embryos derived from parthenogenetic (PA) activation comprise exclusively of maternal genomes and represent an important biological model for studies on epigenetic processes, including DNA methylation^1^. However, due to the absence of paternal gene expression and aberrant methylation profile, embryos generated from PA have a developmental failure^2^. Accumulating studies have also demonstrated abnormal DNA methylation in PA embryos; however, there is a paucity of studies related to the role of RNA methylation in PA embryos^3,4^.

*N*^6^-methylation of adenosine (*N*^6^-methyladenosine or m^6^A), is the most abundant post-transcriptional modification of RNA^5,6^. An increasing number of studies indicate that m^6^A plays a crucial role in several key developmental processes of the cell as well as regulates several aspects of RNA processing mechanisms that control gene expression in diverse physiological processes. Furthermore, the effects of m^6^A on RNA are predominantly determined by the dynamic interplay between its methyltransferases (“writers”), binding proteins (“readers”), and demethylases (“erasers”). The insulin-like growth factor-2 mRNA-binding protein 1 (*Igf2bp1*) as novel m^6^A “reader,” recognizes m^6^A sites on target mRNAs and regulates the mRNAs’ fate^7–9^. Recent studies have identified *Igf2bp1* as a conserved ‘oncogenic’ m^6^A-reader which plays essential roles in carcinogenesis. Moreover, Igf2bp proteins could enhance mRNAs stability and translation through m6A modification^8^. These pieces of evidence indicate that *Igf2bp1* expression is associated with m6A modification and might have a role in PA embryonic development.

MicroRNAs (miRNAs) are endogenous small non-coding RNAs of approximately 22 nucleotides in length which are involved in the posttranscriptional regulation of gene expression by the degradation of their target mRNAs and/or inhibiting their translation. Recently, miR-670 has been reported to target INTS4 in the glomeruli of NZB/W F1 mice with lupus nephritis^10^. Moreover, miR-670 has been identified to be associated with endometrial cancer by targeting KCNS1^11^. Betaine (*N,N,N*-trimethylglycine), as a major methyl donor in the cycle of one-carbon metabolism, has been extensively used to treat pre-implantation embryos to improve the development of embryos^12^. Recent studies have reported that betaine has a role in mouse oocyte meiotic maturation through choline dehydrogenase^13^. Furthermore, betaine can efficiently restore the development of embryos injured by ethanol through a global level of genome DNA methylation in the mouse embryo^14^. In the present study, the expression levels of *Igf2bp1* and m6A were determined using qRT-PCR during early PA embryonic development following treatment with si-RNA and betaine. Overall, the role of *Igf2bp1* in m6A modification was analyzed in the development of PA embryos.

## Results

### Knockdown of *Igf2bp1* expression attenuated global mRNA m6A expression levels

**Figure 1:**
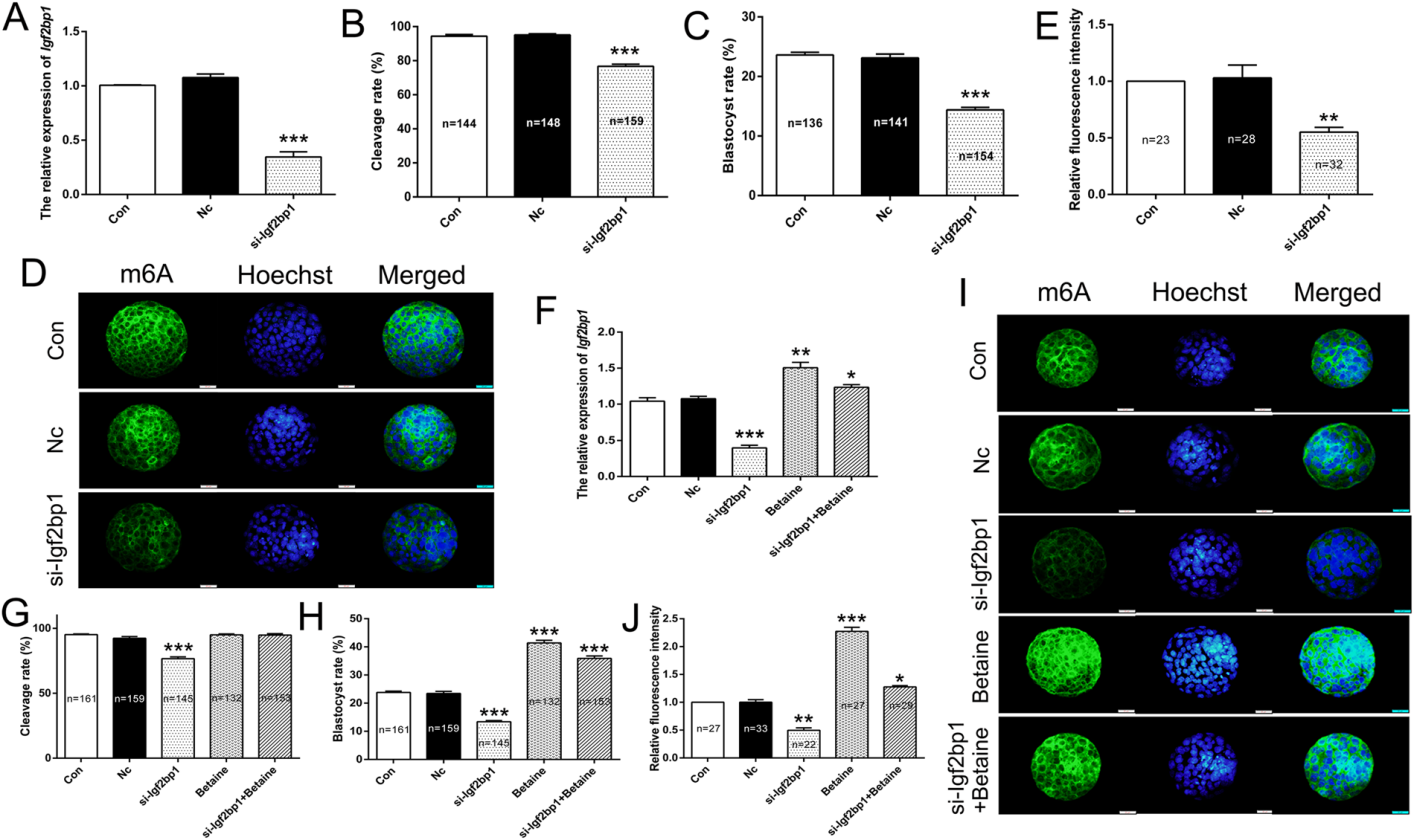
The expression of *Igf2bp1* and m6A in PA embryos. Relative expression levels of *Igf2bp1* as analyzed by qRT-PCR following micro-injection with si-Igf2bp1 in PA embryos (A). (**A**) Statistical analyses of cleavage (**B**) and blastula (**C**) rates following micro-injection with si-Igf2bp1. Immunofluorescence localization of m6A following micro-injection with si-Igf2bp1 (D). (**D**) Statistical analyses of fluorescence intensity (E). (**E**) Relative expression levels of *Igf2bp1* as analyzed by qRT-PCR after treatment with betaine in PA embryos (F). (**F**) Statistical analyses of cleavage (**G**) and blastula (**H**) rates following treatment with betaine. Immunofluorescence localization of m6A following treatment with betaine (I). (**I**) Statistical analyses of fluorescence intensity (J). (**J**) The data are represented as the mean ± SD (n = 3). Green, indicates m6A. Blue, indicates Hoechst. The bar = 20 μm. *p < 0.05, **p < 0.01 and ***p < 0.005 indicate statistically significant differences.

To investigate the role of *Igf2bp1* during embryonic development, si-Igf2bp1 and negative control siRNA were microinjected into a zygote. The expression levels of *Igf2bp1* mRNA were analyzed using qRT-PCR. The results indicated that *Igf2bp1* expression levels were significantly downregulated in PA embryos injected with si-Igf2bp1 compared with those in the negative control siRNA-injected embryos (Nc) or non-injected embryos (Con) (Fig. 1A). Moreover, knockdown of *Igf2bp1* expression significantly suppressed the cleavage and blastula rates in PA embryos (Fig. 1B,C). Furthermore, m6A expression level was analyzed using immunofluorescence (IF). The results indicated that m6A expression was significantly suppressed in PA embryos injected with si-Igf2bp1 compared with the Nc group and Con group (Fig. 1D,E). Collectively, these findings suggested that *Igf2bp1* markedly affected the global mRNA levels of m6A and *Igf2bp1* expression were associated with PA embryo development.

### Betaine up-regulated m6A expression and promoted PA embryonic development

To investigate whether the blastocyst rate was associated with *Igf2bp1* expression, the betaine was applied to the culture of PA embryos. qRT-PCR results revealed that betaine significantly upregulated the expression of *Igf2bp1* following transfection with si-Igf2bp1 (Fig. 1F). Furthermore, the cleavage rate was restored after treatment with betaine (Fig. 1G). Besides, the blastula rate was also noticeably increased in the si-Igf2bp1 group after treatment with betaine (Fig. 1H). In addition, the results of IF showed that betaine significantly up-regulated the expression of m6A (Fig. 1I,J). Together, these results indicated that increased expression levels of *Igf2bp1* and global expression of m6A promoted PA embryonic development following treatment with betaine.

### miR-670-3p regulated *Igf2bp1* expression in PA embryos apoptosis

**Figure 2:**
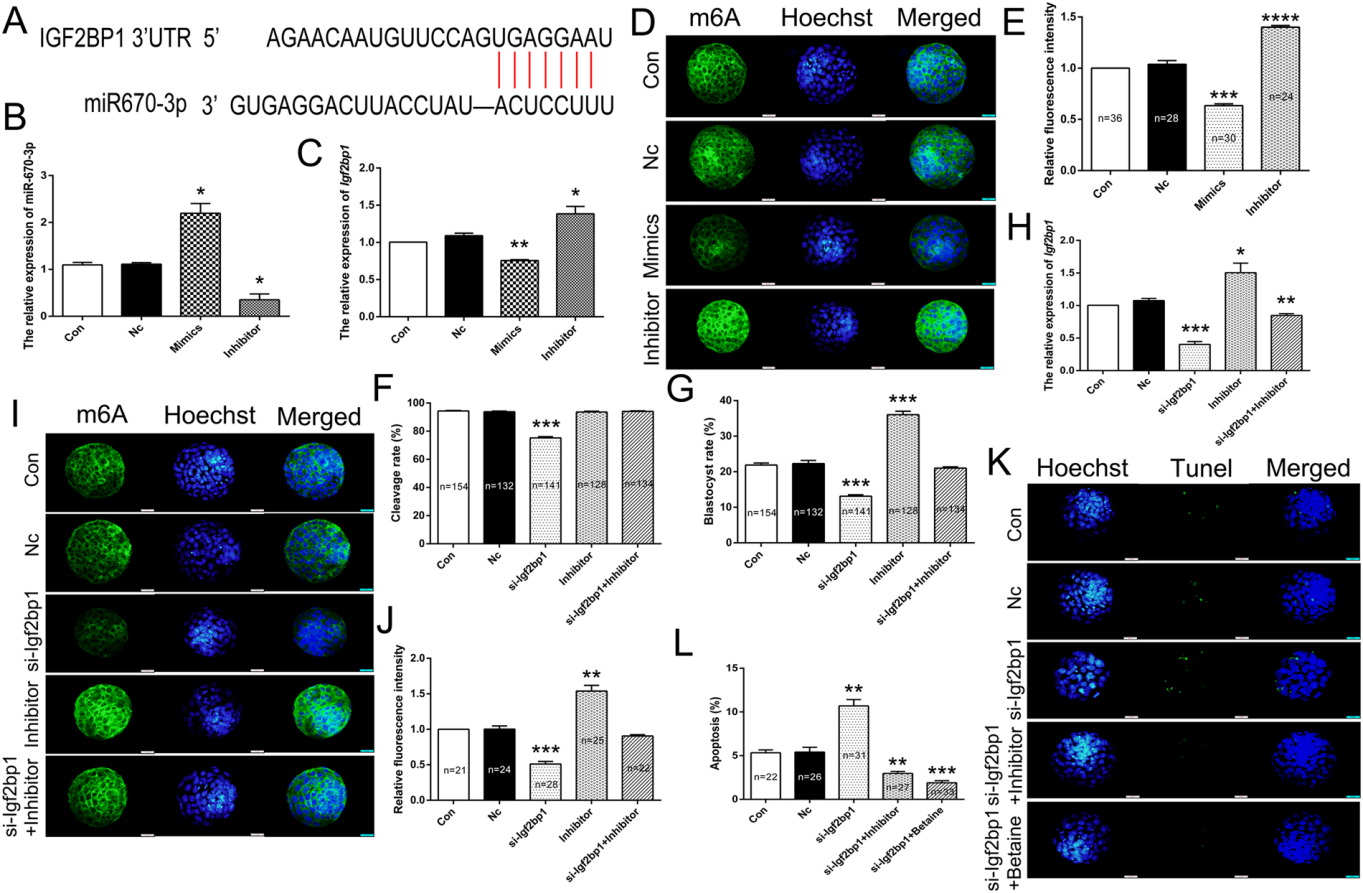
The expression of *Igf2bp1* was regulated by miRNA-670. Schematic representations of target sites of miRNA-670 within 3′UTR of *Igf2bp1* mRNA (A). (**A**) Relative expression levels of miRNA-670 (**B**) and *Igf2bp1* (**C**) as analyzed by qRT-PCR. Immunofluorescence localization of m6A following micro-injection with miRNA-670 mimics and inhibitor (D). (**D**) Statistical analysis of fluorescence intensity (E). (**E**) Statistical analysis of cleavage (**F**) and blastula (**G**) rates following micro-injection with a miRNA-670 inhibitor in PA embryos. Relative expression levels of *Igf2bp1* as analyzed by qRT-PCR (**H**). Immunofluorescence localization of m6A following micro-injection with miRNA-670 inhibitor and si-Igf2bp1 (I). (**I**) Statistical analysis of fluorescence intensity (J). (**J**) Green, indicates m6A. Blue, indicates Hoechst in m6A IF staining. TUNEL assay to analyze the cell apoptosis in PA embryos (K). (**K**) Statistical analysis of cell apoptosis (L). (**L**) Blue, indicates Hoechst. Green, indicates Tunel. *p < 0.05, **p < 0.01, ***p < 0.005 and ****p < 0.001 indicate statistically significant differences.

To investigate the post-transcriptional regulation of *Igf2bp1*, potential miRNAs targeting *Igf2bp1* were identified using bioinformatics tools (Fig. 2A). The qRT-PCR analysis revealed that miR-670-3p could negatively regulate the expression of *Igf2bp1* (Fig. 2B,C). Besides, IF results indicated that inhibitors of miR-670-3p markedly augmented m6A expression levels compared to the Nc group (Fig. 2D,E). Moreover, the cleavage and blastula rates were analyzed in the si-Igf2bp1 group following transfection with an inhibitor of miR-670-3p. The results revealed that the miR-670-3p inhibitor could restore the cleavage and blastula rates in PA embryos (Fig. 2F,G). To verify whether *Igf2bp1* is a direct target of miR-670-3p, the expression of *Igf2bp1* in the si-Igf2bp1 group was analyzed following treatment with miR-670-3p inhibitor. The qRT-PCR analysis indicated that *Igf2bp1* expression was markedly up-regulated in the si-Igf2bp1 group following treatment with miR-670-3p inhibitor (Fig. 2H). Furthermore, IF data suggested that miR-670-3p inhibitor could increase m6A expression levels in the si-Igf2bp1 group (Fig. 2I,J). Besides, the TUNEL assay revealed that treatment with miR-670-3p inhibitor noticeably reduced the number of apoptotic cells (Fig. 2K,L). Collectively, these results indicated that miR-670-3p modulated the expression of *Igf2bp1* and influenced m6A expression levels.

## Discussion

Our previous study suggested that global mRNA m6A expression levels are associated with PA embryonic development^15^. Therefore, to further elucidate the underlying molecular mechanism of m6A modification in mice parthenogenesis, the expression of *Igf2bp1* was analyzed at the cleavage and blastocyst stages of the PA embryos in the present study. The results indicated that an increased rate of development failure is associated with reduced m6A expression levels in PA embryos. These findings confirmed that m6A expression level was crucial for embryonic development which affects RNA stability. Moreover, *Igf2bp1* exhibits a role in recognizing and binding to the m6A writing sites of targeted mRNAs^16^. This implied that reduced expression of *Igf2bp1* might inhibit m6A level which is in concordance with our data. Thus, the findings of the present study suggested that the *Igf2bp1* exhibits the ability to regulate PA embryonic development through m6A modification.

Further, to investigate the post-transcriptional regulation of *Igf2bp1*, potential miRNAs targeting *Igf2bp1* were identified using bioinformatics tools. To confirm that *Igf2bp1* is the target of miR-670, mimics, and miR-670 inhibitor was micro-injected in the PA embryos. The results indicated that in the presence of the miR-670 inhibitor, *Igf2bp1* expression was markedly upregulated and the expression levels of m6A were considerably promoted as compared with the si-Igf2bp1 group. Moreover, the inhibitor of miR-670 rescued the development of embryos which was suppressed by the loss of expression of *Igf2bp1*. Indeed, several miRNAs have been recognized to play a crucial role in the development of PA embryos^17,18^. Thus, considering these pieces of evidence, we speculated that m6A methylation level is associated with miR-670 and *Igf2bp1* and may represent crucial factors for developmental failure.

Furthermore, treatment with betaine promotes DNA methylation^19,20^. Moreover, there is evidence that betaine improves embryo development in mice^13^. However, there is a paucity of literature regarding the role of m6A modification following treatment with betaine. Our result suggested that the m6A level was up-regulated following treatment with betaine. Furthermore, betaine rescued *Igf2bp1* expression and promoted the development of PA embryos in the si-Igf2bp1 group. These findings indicated that m6A modification which was regulated by betaine exhibited a crucial role in PA embryonic development. In addition, compared to the si-Igf2bp1 group, treatment with betaine and miR-670 inhibitor markedly inhibited apoptosis. Previous studies suggested that *Igf2bp1* overexpression suppressed apoptosis in HTR-8/SVneo cells which were consistent with our results^21^. Thus, *Igf2bp1* expression exhibits a crucial role in cell apoptosis in PA embryonic development.

## Conclusion

The findings of this study suggested that loss of expression of *Igf2bp1* markedly suppressed PA embryonic development and m6A levels. Furthermore, our data also demonstrated that miR-670 inhibitor augmented the expression of *Igf2bp1* and rescued cleavage and blastula rates. Besides, betaine could enhance the m6A methylation level in PA embryos. In conclusion, miR-670 functions as the regulator of *Igf2bp1* expression and plays a crucial role in PA development through m6A modification.

## Methods

### Ethics statement

The animals were cared for in accordance with the Guide for the care and use of laboratory animals in China. All experimental procedures were approved by the Animal Care and Use Committee of Jilin University, Changchun, China (Grant No. 201706005).

### Production of PA embryos

The female ICR mice (6–8 weeks old) were acquired from the School of Medical Science, Jilin University. Superovulation was induced in female mice by an intraperitoneal administration of 10 IU pregnant mare’s serum gonadotropin (PMSG; Merck Millipore) and was injected with 10 IU human chorionic gonadotropin (hCG; Sigma-Aldrich, St. Louis, MO, USA)) 48 h later. PA embryos production was performed according to the procedure described by us previously. Briefly, the oviducts of female mice were carefully removed, and cumulus-oocyte complexes (COCs) were extracted from the oviducts as unfertilized oocytes after post-PMSG and -hCG injection. Oocytes were then denuded from their cumulus cells by briefly exposing the oocytes at the metaphase stage of the second meiotic division (MII) to a serum-free medium comprising hyaluronidase (Sigma). The oocytes were then treated with a calcium ionophore (ionomycin calcium salt; Sigma) for 5 min. Parthenogenetic activation was achieved by incubating the unfertilized oocytes in M16 medium supplemented with 6-DMAP (2 mmol/l; Sigma) for 4 h. Subsequently, these unfertilized oocytes were transferred to the fresh M16 medium. PA activation was established by the presence of two pronuclei, which developed to the two-celled stage. The embryo culture was conducted until the blastocyst stage.

### Microinjection of microRNA and siRNA into the zygote

Before microinjection, oocytes were cultured to metaphase-II (MII) with completed parthenogenetic activation. Microinjections for mouse miR-670 (mmu-miR-670-3p) and si-Igf2bp1 into the cytoplasm of zygote were performed using an Eppendorf Cell Tram Vario system (Eppendorf, Hamburg, German) and a Nikon TE2000-U inverted microscope (Nikon, Tokyo, Japan). Similarly, the siRNA negative control and miRNA negative control was microinjected into zygote to serve as the negative control, while non-injected zygote was used as the normal control. The final concentration of the control or miR-670 or si-Igf2bp1 was 20 μM. The injected zygotes were cultured M16 medium until the blastocyst stage. The sequence of si-Igf2bp1 was (5′ to 3′): TTTACTTCCTCCTTGGGACTT; the sequence of mmu-miR-670-3p mimics was (5′ to 3′): UUUCCUCAUAUCCAUUCAGGAGU; the sequence of mmu-miR-670-3p inhibitor was (5′ to 3′): ACACUCCUGAAUGGAUAUGAGGAAA; the sequence of miRNA Nc was (5′ to 3′): UUCUCCGAACGUGUCACGU.

### Gene expression analysis

The gene expression analysis was performed as described previously^22^. Briefly, the total RNA was extracted from embryos of each group (n = 50). Quantitative real-time PCR (qRT-PCR) was used to analyze the expression of *Igf2bp1*. qRT-PCR was performed using the following two-step cycling program: initial denaturation at 95 °C for 3 min, followed by 40 cycles of denaturation at 95 °C for 10 s, annealing at 60 °C for 15 s, and extension at 72 °C for 30 s. The 2^−ΔΔCT^ method was used to determine the relative gene expression, which was normalized to the expression of endogenous control, *Gapdh*. The experiments were performed at least in triplicate. The primer sequences used in the qRT-PCR analysis were as follows: *Igf2bp1* forward, 5′-ATCGGAGCTGAGGTGGAATA-3′; reverse, 5′-CTCGGGGAAAGTAGAACTGC-3′; *Gapdh* forward, 5′-AGGTCGGTGTGAACGGATTTG-3′; reverse, 5′-TGTAGACCATGTAGTTGAGGTCA-3′.

### Immunofluorescence staining

The mouse immunofluorescence (IF) staining was performed on oocytes/embryos, as described previously^15^. Briefly, using Tyrode’s Solution (Jisskang, China), the thinning of zona pellucida was performed. Then, embryos were fixed with 4% paraformaldehyde in phosphate-buffered saline (PBS) for 30 min at room temperature (RT). Following fixation, the embryos were permeabilized with 0.2% Triton X-100 in PBS for 30 min. They were then blocked in PBS containing 1% bovine serum albumin (BSA) for 1 h. Next, the embryos were probed with m6A (1:500, Abcam) antibodies and incubated at 4 °C overnight. After incubation with the primary antibody, embryos were washed three times with PBS followed by incubation with Alexa Fluor 488 conjugated secondary (anti-rabbit) antibodies for 1 h at RT. The embryos were stained with 10 ng/ml Hoechst 33342 (Thermo Scientific) for 15–20 min. Slides were mounted using an anti-fade mounting medium (BOSTER, China). Images were acquired analyzed using a fluorescence microscope 1–2 h after staining. The exposure time of IF staining was 10 ms for Hoechst and 70 ms for m6A. The average fluorescence intensities were analyzed using ImageJ analysis software^23^.

### TUNEL assay

Briefly, the blastocysts were fixed with 4% paraformaldehyde for 1 h at RT. After fixation, the blastocysts were permeabilized with 0.1% Triton X-100 for 1 h at 37 °C. The blastocysts were blocked with PBS containing 1% BSA in the dark for 1 h at 37 °C with TdT and fluorescein-conjugated dUTP (*In Situ* Cell Death Detection kit; Roche, Germany). The blastocysts were stained with 10 μg/mL Hoechst 33342 for 15 min. Slides were mounted using an anti-fade mounting medium (BOSTER, China). The blastocysts were mounted on a coverslip and a glass slide using an antifade mounting medium (BOSTER, China). Images were acquired using a fluorescence microscope 1–2 h after staining and the cell apoptotic rates were analyzed using ImageJ software.

### Statistical analysis

TargetScan program (www.targetscan.org) was used to predict miRNAs (miR-670) binding sites on mRNA of *Igf2bp1*. Data are presented as mean ± SD of at least three replicates. Differences between groups were assessed using Student’s *t*-test (Unpaired *t*-test). Statistical analyses were performed with GraphPad Prism 5.0 (GraphPad Software, Inc., San Diego, CA). A p-value of <0.05 was considered statistically significant.

## Supplementary information

Supplementary information.
