# Supplementary material for: microRNA-670 modulates Igf2bp1 expression to regulate RNA methylation in parthenogenetic mouse embryonic development
Source: Sci Rep. 2020 Mar 16;10:4782. doi: 10.1038/s41598-020-61816-3 (PMC7076016; doi:10.1038/s41598-020-61816-3)
Supplement: Supplementary file 1 — Supplementary information. [file 41598_2020_61816_MOESM1_ESM.pdf]

microRNA-670 modulates *Igf2bp1* expression to regulate RNA methylation in parthenogenetic mouse embryonic development

Jindong Hao [a, 1], **Haobo Hu** [a, 1], **Ziping Jiang** [b, 1], **Xianfeng Yu** [a], **Chengshun Li** [a], **Lin Chen** [a], **Yidan Xia** [b], **Da Liu** [c, *], **Dongxu Wang** [a, *]

[a] *Laboratory Animal Center, College of Animal Science, Jilin University, Changchun, China*

[b] *Department of hand surgery, The First Hospital of Jilin University, Changchun, China*

[c] *Department of Pharmacy, Changchun University of Chinese Medicine, Changchun, China*

Keywords: miR-670; *Igf2bp1*; m6A; Parthenogenetic; Embryos development

Correspondence: *Da Liu, Department of Pharmacy, Changchun University of Chinese Medicine, 1035#, Boshuo Road, Changchun 130117, China, Tel.: (86) 431-86172211; E-mail: liuda_1986@163.com. Dongxu Wang, College of Animal Science, Jilin University, 5333#, Xi'an Road, Changchun 130062, China, Tel.: (86) 431-87836175; Fax: (86) 431-87980131; E-mail: wang_dong_xu@jlu.edu.cn.*

[1]*The authors Contributed equally to this work*

Author Contribution

Dongxu Wang designed the experiments and wrote the manuscript. Jindong Hao, Haobo Hu and Chengshun Li performed cell experiment and gene expression analysis. Da Liu, Ziping Jiang and Xianfeng Yu contributed reagents and materials. Lin Chen, Yidan Xia carried out animal experiment. Da Liu analyzed the data and prepared figures. All authors reviewed the manuscript.

Competing interests

The authors declare that they have no competing interests.

Table S1 Primers for qRT-PCR analysis

Genes	Annealing (°C)	Primer sequences (5’→3’)	Reference/accession
Igf2bp1	60	F: ATCGGAGCTGAGGTGGAATA<br>R: CTCGGGGAAAGTAGAACTGC	[1]
Gapdh	60	F: AGGTCGGTGTGAACGGATTTG<br>R: TGTAGACCATGTAGTTGAGGTCA	
miR-670-3P	60	F:ACACTCCAGCTGGGTTTCCTCATATC<br>CATTCAG<br>R: CAGTGCGTGTCGTGGAGT	
U6	60	F:GCTTCGGCAGCACATATACTAAAAT<br>R: CGCTTCACGAATTTGCGTGTCAT	

References

1 Nguyen, L. H. *et al.* Lin28b Is Sufficient to Drive Liver Cancer and Necessary for Its Maintenance in Murine Models. *Cancer cell* **26**, 248-261, doi:10.1016/j.ccr.2014.06.018 (2014).
